# Correction: Cutting-edge novel device in the treatment of obesity

**DOI:** 10.1055/a-2288-7804

**Published:** 2024-03-20

**Authors:** Jan Kral, Jana Selucka, Katerina Waloszkova, Marek Buzga, Julius Spicak, Evzen Machytka

**Affiliations:** 1 Institute for Clinical and Experimental Medicine, Hepatogastroenterology Department, Prague, Czech Republic; 2 Department of Internal Medicine, Second Faculty of Medicine, Charles University, Prague, Czech Republic; 3 Department of Physiology and Pathophysiology, Faculty of Medicine, University of Ostrava, Ostrava, Czech Republic

**Correction**

**Correction: Cutting-edge novel device in the treatment of obesity** Kral J, Selucka J, Waloszkova K et al. Cutting-edge novel device in the treatment of obesity. Endoscopy 2023; 55: E1124–E1125, doi:10.1055/a-2173-7560 In the above-mentioned article, the statement for the conflict of interest has been corrected. Correct is: The E-Video was supported by Nitinotes, the company that developed this device. This was corrected in the online version on March 19, 2024.

